# Innovative Alternative Technologies to Extract Carotenoids from Microalgae and Seaweeds

**DOI:** 10.3390/md14110214

**Published:** 2016-11-22

**Authors:** Mahesha M. Poojary, Francisco J. Barba, Bahar Aliakbarian, Francesco Donsì, Gianpiero Pataro, Daniel A. Dias, Pablo Juliano

**Affiliations:** 1 Discipline of Laboratory Medicine, School of Health and Biomedical Sciences, RMIT University, 3083 Bundoora, Australia; mahesha.poojary@unicam.it (M.M.P.); daniel.dias@rmit.edu.au (D.A.D.); 2 Chemistry Section, School of Science and Technology, University of Camerino, via S. Agostino 1, 62032 Camerino, Italy; 3 Nutrition and Food Science Area, Preventive Medicine and Public Health, Food Sciences, Toxicology and Forensic Medicine Department, Faculty of Pharmacy, Universitat de València, Avda. Vicent Andrés Estellés, s/n, 46100 Burjassot, València, Spain; 4 Department of Civil, Chemical and Environmental Engineering, Pole of Chemical Engineering, University of Genoa, via Opera Pia 15, 16145 Genoa, Italy; Bahar.Aliakbarian@unige.it; 5 Department of Industrial Engineering, University of Salerno, via Giovanni Paolo II 132, 84084 Fisciano, Italy; fdonsi@unisa.it (F.D.); gpataro@unisa.it (G.P.); 6 ProdAl Scarl, via Ponte don Melillo, 84084 Fisciano, SA, Italy; 7 CSIRO Agriculture and Food, 671 Sneydes Road, 3030 Werribee, VIC, Australia; Pablo.Juliano@csiro.au

**Keywords:** marine microalgae, seaweeds, carotenoids, nonconventional extraction, electrotechnologies, pulsed electric field-assisted extraction, supercritical fluid extraction, green processing, microwave-assisted extraction, marine drugs

## Abstract

Marine microalgae and seaweeds (microalgae) represent a sustainable source of various bioactive natural carotenoids, including β-carotene, lutein, astaxanthin, zeaxanthin, violaxanthin and fucoxanthin. Recently, the large-scale production of carotenoids from algal sources has gained significant interest with respect to commercial and industrial applications for health, nutrition, and cosmetic applications. Although conventional processing technologies, based on solvent extraction, offer a simple approach to isolating carotenoids, they suffer several, inherent limitations, including low efficiency (extraction yield), selectivity (purity), high solvent consumption, and long treatment times, which have led to advancements in the search for innovative extraction technologies. This comprehensive review summarizes the recent trends in the extraction of carotenoids from microalgae and seaweeds through the assistance of different innovative techniques, such as pulsed electric fields, liquid pressurization, supercritical fluids, subcritical fluids, microwaves, ultrasounds, and high-pressure homogenization. In particular, the review critically analyzes technologies, characteristics, advantages, and shortcomings of the different innovative processes, highlighting the differences in terms of yield, selectivity, and economic and environmental sustainability.

## 1. Introduction

Carotenoids are a class of terpenoid pigments with a tetraterpenes (C_40_) backbone, responsible for a range of colors, such as brilliant yellow, orange and red in fruits, vegetables, and aquatic creatures [1]. They contain highly conjugated polyene chromophoric chains which give rise to distinct colors and functions [2], constituting two major classes of molecules: (i) carotenes, which are strictly hydrocarbons (e.g., α-carotene, β-carotene, and lycopene) and (ii) xanthophylls, which are similar to carotenes but contain oxygen (e.g., lutein, zeaxanthin, neoxanthin, violaxanthin, flavoxanthin, and fucoxanthin). Carotenoids are predominantly found in plants; however, they are also present in many algae, bacteria and some fungi and play a key role in light harvesting and photo protection in photosynthetic organisms [3]. To date, over 600 unique carotenoids have been identified [4].

In recent decades, there has been a considerable amount of evidence supporting the role of carotenoids as food colorants and antioxidants with beneficial effects on human health, especially with regards to the prevention of chronic diseases, particularly certain cancers, cardiovascular and eye diseases [5,6,7]. They have been widely used as nutraceuticals, cosmeceuticals and feed supplements in aquaculture sectors [8,9]. For this reason, the global demand for carotenoids is growing remarkably with the worldwide carotenoids market estimated at USD 1.24 billion in 2016 and is expected to reach USD 1.53 billion by 2021 [10]. Currently, the major commercial carotenoids are produced by chemical synthesis [11]; however, in recent year, the increasing consumers’ concerns for public health, safety and environmental burden have driven the growth of the market demand for natural carotenoids-based products.

Marine microalgae and seaweeds serve as a unique, sustainable and alternative source of carotenoids [9,12]. Therefore, in recent years, the industrial interest towards the production of natural carotenoids using algae has considerably increased, as they offer cost, scale, time and yield advantages over terrestrial plants.

In microalgae, carotenoids can be classified into two groups, primary and secondary carotenoids, based on their metabolism and function. Primary carotenoids are structural and functional components in the photosynthetic apparatus, which take direct part in photosynthesis. Secondary carotenoids refer to extra-plastidic pigments produced in large quantities, through carotenogenesis, after exposure to specific environmental stimuli [13,14]. Primary and secondary carotenoids are of considerable interest as natural colorants as well as their potential in human health. Specifically, they possess a wide range of distinctive biological activities, including antioxidant, cardiovascular protection, anticancer, antidiabetic, and anti-obesity, which have been recently reviewed [15]. The primary microalgae carotenoids include α-carotene, β-carotene, lutein, fucoxanthin, violaxanthin, zeaxanthin, and neoxanthin, among others. Examples of secondary carotenoids, include: astaxanthin, canthaxanthin, and echinenone [16]. Interestingly, among these compounds, astaxanthin, zeaxanthin, β-carotenes, fucoxanthin and lutein are commercially important carotenoids, which are widely found in marine microalgae.

Seaweeds (macroalgae) also serve as an important source of carotenoids [17], such as fucoxanthin, lutein, β-carotene and siphonaxanthin. In particular, fucoxanthin is a characteristic orange xanthophyll, which is abundant in several brown seaweeds including *Undaria pinnatifida* [18,19,20,21,22,23], *Hijikia fusiformis* [24], *Laminaria japonica* [21,25,26], *Sargassum* sp. [27,28,29,30,31,32], and *Fucus* sp. [33]. It is one of the most abundant carotenoids in seaweeds, accounting for more than 10% of the estimated total natural production of carotenoids [34], with remarkable biological properties, including anticancer, anti-inflammatory, antiobesity and neuroprotective bioactivities [34,35,36,37,38].

**Figure 1:**
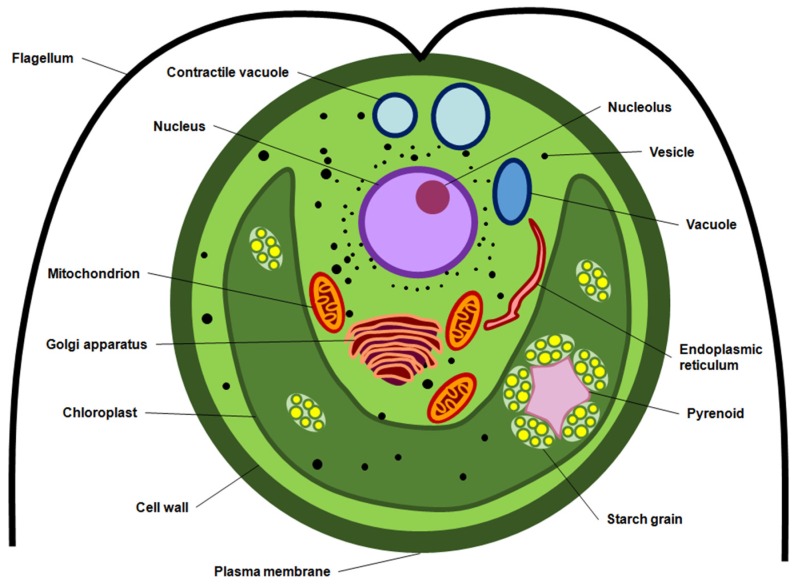
Illustration detailing organelles present in a typical marine unicellular microalgae.

Carotenoids produced by algae are generally localized in the chloroplast or accumulated in vesicles, cytoplasmic matrix or bound to membranes and other macromolecules in the intracellular space. The cell wall and plasma membrane surrounding the cell, as well the chloroplast membranes, act as a barrier which greatly limits the rate of mass transfer of carotenoids and other intracellular compounds during conventional extraction processes. Figure 1 illustrates a marine microalga, highlighting that the extraction of carotenoid yield requires the disruption or permeabilization of the cell wall, of the plasma membrane and, depending on the biological features (e.g., organelle localization), eventually of the chloroplast membrane. Algae display complex cell envelope structures and their composition varies from species to species [39]. Therefore, it is essential to develop and optimize efficient methods for the selective extraction of these compounds, which takes into account the biological diversity as well as the localization of carotenoids within specific organelles.

Generally, carotenoids are recovered from microalgae and seaweeds by means of conventional solvent extraction (e.g., Soxhlet extraction) using organic solvents [40]. However, these methods are time-consuming, and often require the usage of relatively large amounts of solvents, which is expensive and not environmental friendly. The use of innovative non-conventional techniques, based on the physical membrane permeabilization or lysis, to selectively or non-selectively increase the rate of mass transfer of carotenoids from the intracellular space of microalgae and seaweeds, has gained growing interest in recent years. In particular, this review extensively details the recent advances in the use of novel technologies to recover carotenoids from microalgae and seaweeds, including electrotechnologies-assisted extraction, such as pulsed electric field (PEF), moderate electric field (MEF), high-voltage electric discharges (HVED), as well as supercritical fluid extraction (SFE), subcritical fluid extraction, pressurized liquid extraction (PLE), microwave-assisted extraction (MAE), ultrasound-assisted extraction (UAE) and high pressure homogenization (HPH) treatment. These alternative technologies have several advantages, including rapid extraction (e.g., PLE, MAE, UAE, PEF, MEF, HVED and HPH), low solvent consumption (e.g., PEF, PLE, MAE and UAE), use of “green” environmentally friendly solvents (e.g., SFE), superior recovery (e.g., MAE, subcritical fluid extraction, HVED, UAE and HPH) and higher selectivity (e.g., PEF and SFE).

## 2. Extraction Technologies for Carotenoids

### Conventional Extraction Methods

Conventional extraction of algae intracellular products is typically conducted from dry biomass and is based on maceration and thermal extraction using organic or aqueous solvents, depending on the polarity of the target compounds to be extracted. Carotenoids exhibit varying polarities, solubilities and chemical stabilities. Therefore, a suitable solvent system must be selected on the basis of the target carotenoids, which could selectively and efficiently extract carotenoids with greater purity. Since most carotenoids possess a high degree of hydrophobicity, their effective extractions requires the use of non-polar solvents, for example *n*-hexane, dichloromethane, dimethyl ether, diethyl ether etc. [23,41]. Acetone, octane, biphasic mixtures of several organic solvents have also been studied for the selective extraction of carotenoids [42,43,44]. Recently, the use of several green solvents such as ethanol, limonene and biphasic mixtures of water and organic solvents have been investigated for recovery of carotenoids from microalgae [45,46]. However, extraction efficiency, selectivity and high solvent consumption still remain a limiting factor in the conventional solvent extraction process. One avenue to overcome this problem can be based on the usage of a multi-stage extraction procedure eventually assisted by different physical and chemical methodologies which may selectively target the desired intracellular carotenoids.

## 3. Nonconventional Extraction of Carotenoids

### 3.1. Electrotechnologies

**Figure 2:**
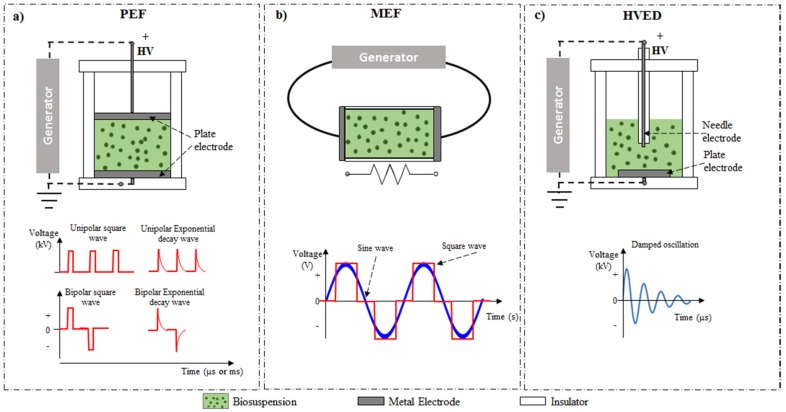
Schematics of typical experimental set-up and pulse protocols for (**a**) pulsed electric field (PEF)-assisted extraction; (**b**) moderate electric field (MEF)-assisted extraction; and (**c**) high voltage electric discharge (HVED)-assisted extraction.

In recent years, there has been an emerging interest in the use of electrotechnologies, such as PEF, MEF, and HVED as a non-thermal, green extraction techniques for targeting intracellular compounds from plant or biosuspensions [47,48,49,50]. As illustrated in Figure 2, although each of these electrotechnologies has its own mode of treatment and mechanism of delivering electrical current through the processed biomaterial, they all induce a certain degree of cell disintegration allowing for the selective extraction of intracellular compounds. The principles and recent advances in application of electrotechnologies to extract carotenoids from microalgae and seaweeds are described in the following subsections.

#### 3.1.1. Pulsed Electric Field (PEF)-Assisted Extraction

PEF processing is a non-thermal technique, which has received increasing attention in recent years [51]. In PEF-assisted extraction, the sample matrix is placed between two electrodes in a batch or a continuous flow treatment chamber and exposed to repetitive electric frequencies (Hz–kHz) with an intense (0.1–80 kV/cm) electric field for very short periods (from several nanoseconds to several milliseconds). The pulses commonly used in PEF treatments are unipolar or bipolar, with either exponential or square-wave shaped frequencies (Figure 2a). The application of electric pulses causes the formation of reversible or irreversible pores in the cell membranes, defined as electroporation or electropermeabilization, which consequently aids the rapid diffusion of the solvents and the enhancement of the mass transfer of intracellular compounds [51]. The selective extraction of analytes can be achieved by controlling the pore formation, which is dependent on the intensity of the treatment applied (electric field strength, single pulse duration, treatment time or total specific energy input) and the cell characteristics (i.e., size, shape, orientation in the electric field) [52].

Recently, a few authors have investigated the ability of PEF to enhance the extractability of carotenoids from microalgae obtaining controversial results (Table 1). A PEF pretreatment at 15 kV/cm and 100 kJ/kg increased the extraction of carotenoids from marine microalgae *Chlorella vulgaris* and *Spirulina platensis* up to 525 and 150%, respectively, compared to the conventional ball milling homogenization process alone [53]. Subsequently, a PEF treatment at 20 kV/cm electric field strength with an energy density of 13.3–53.1 kJ/kg for 1–4 ms did not increase the carotenoids yield extracted from marine microalga *Nannochloropsis* sp. [54]. It is likely that the use of a polar solvent, such as water, together with the thick cell wall structure of *Nannochloropsis* sp., with the presence of secondary structures, complex polysaccharide networks and an outer algaenan layer [55] could be the reasons for the observed scarce efficiency of PEF treatments. Furthermore, extraction of carotenoids which are bound to chloroplasts requires more intense treatments, as the chloroplast membranes has to be electroporated along with the plasma membrane to improve the mass transfer of extractable compounds. Considering that the external electric field threshold required to trigger electroporation is inversely related to cell size [56,57], higher electric field strengths are required for permeabilization of smaller internal organelles like chloroplasts [58]. Specifically, nanosecond pulses with electric field strengths around 100 kV/cm have been reported to cause the electroporation of intracellular organelles [59]. However, more recently, it has been demonstrated that also longer pulses of lower intensity, currently used for the electroporation of plasma membranes, can also cause non-thermal intracellular effects, including organelles electroporation [58].

In a recent study, it has been found that PEF treatments in the microsecond range (3 μs pulse duration) with a field strength in the range of 20–25 kV/cm can cause significant, irreversible electroporation of *C. vulgaris,* resulting in improved carotenoid yield. Lower electric field strengths (10 kV/cm) in the microsecond range resulted only in the reversible electroporation, leading to lower extraction yields [60]. However, the lower degree of electroporation at lower electric field strengths can be compensated by increasing the treatment duration to the milliseconds range [61], at the expense of a specific energy higher than that of treatment at higher electric field strengths in the microsecond range [61]. Interestingly, when the extraction of carotenoids was performed after 1 h of PEF treatment, the authors noted that the yield increased up to 1.58 mg/L of culture for the PEF treated samples in the microsecond range, while no further increase was observed for the sample treated in the millisecond range [61]. Nevertheless, the authors did not find an increase in the degree of permeabilization in the PEF treated cells during the incubation period. The increase in the yield was, therefore, attributed to subsequent plasmolysis of the chloroplast during the incubation time due to osmolytic disequilibrium in the cytoplasmatic space, which facilitated the diffusion of both the solvent into the chloroplast and the carotenoid pigments towards the cytoplasm [61].

The application of multi*-*step extraction procedures, based on the combination of PEF and solvent extractions at various pH and the usage of biphasic mixture of organic solvents, can also assist in the recovery of low water solubility carotenoids with optimum yields [62,63]. Using this approach, Parniakov et al. [63] observed a noticeable increase in the concentrations of carotenoids in the aqueous extracts from *Nannochloropsis* spp. after PEF was applied at pH 8.5, followed by extraction at pH 11. In a further study, the authors efficiently recovered carotenoids and other pigments from *Nannochloropsis* spp. with the application of biphasic mixtures of organic solvents [(i.e., dimethyl sulfoxide (DMSO) and ethanol (EtOH)] and water. A two stage extraction procedure involving PEF treatment (20 kV/cm) of microalgae suspension and extraction in water at the first step, followed by the conventional extraction using biphasic mixtures in the second step, allowed for the efficient extraction of carotenoids in less concentrated mixtures of organic solvents with water [62].

A combination of PEF and moderate thermal treatment could also assist to achieve the required permeabilization effect of rigid algal membrane structures with less severe processing conditions, or to achieve higher efficacy at the same treatment conditions [65]. It was found that a mild thermal treatment enhanced electroporation efficiency of PEF treatment in plant tissues [66,67]. In the case of extraction of luteinfrom *C. vulgaris*, PEF pretreated samples (25 kV/cm for 75 μs) resulted in a 4.5-fold higher concentration of lutein (753 μg/g dw of *C. vulgaris* culture), with respect to to untreated samples, when carried out at 40 °C, whereas the yield increased only of 2.3 and 3.2 fold when carried out at 10 and 25 °C [56]. The increase in yield at higher temperatures was positively correlated with the increase in membrane permeabilization. Furthermore, the yield also increased with the applied electric field strength, which was correlated to the irreversible electroporation of algal membranes. Interestingly, temperature enhanced electroporation under the PEF treatment and decreased the treatment time to achieve the desired yields, consequently reducing the total specific energy required for the treatment [56].

#### 3.1.2. Moderate Electric Field (MEF)-Assisted Extraction

MEF-assisted extraction could also be an attractive alternative method to extract carotenoids from microalgae. MEF can promote cell membrane permeabilization and consequently assists in the diffusion of intracellular compounds from the intracellular matrix (for a detailed principle and mechanism MEF processing, see ref. [68]). The MEF-assisted extraction process involves the application of relatively low electric fields (arbitrarily defined between 1 and 1000 V/cm) in the range of Hz up to tens of kHz, with or without heating, to biomaterials placed between two electrodes [64] (Figure 2b). MEF can cause a wide variety of effects on biological samples depending on the electrical and thermal conditions used in the treatment. Interestingly, in spite of the relatively low field strength applied, it has been shown that MEF treatment can promote at least reversible electroporation of the cell membranes, increasing their permeability [69].

In comparison with PEF, there are only limited data available about the use of MEF in the recovery of intracellular compounds from microalgae (Table 1), although interesting studies have been reported on the extraction of valuable compounds from plant material [49,50]. In a recent study, Nezammahalleh et al. [70] showed that up to 73% of carotenoids (1.21 mg lutein Eq./g sample dw) can be recovered from the *Heterochlorella luteoviridis* microalga biomass using MEF combined with ethanol as solvent (180 V, 60 Hz, 75 mL/100 mL of ethanol solution). In this case, carotenoid extraction yield increased with electrical field strength and ethanol concentration. HPLC*-*UV*-*Vis analysis identified the presence of all*-trans–*lutein (856 μg/g), all*-trans-*zeaxanthin (244 μg/g) and all*-trans-*β*-*carotene (185 μg/g) in major quantities. Besides, all*-trans*-α-carotene, 9-13*-*15*-cis-*β*-*carotene, *cis-*violaxanthin, all*-trans-*violaxanthin and 13-13′*-cis-*lutein were detected in minor quantities [70]. In another study Jaeschke et al. [64] used a two-stage approach to evaluate the effect of MEF pretreatment (0–180 V, 10 min) in the presence of 25 mL/100 mL of ethanol/water solution, followed by the subsequent extraction with ethanol at varying concentrations (25–75 mL/100 mL, 50 min) for the extraction of carotenoids from the microalga *Heterochlorella luteoviridis*. It was observed that the extraction of carotenoids increased as the electrical field strength and ethanol concentration increased, with the highest extraction yields (73%) measured at the maximum values of the two variables (180 V and 75 mL/100 mL of ethanol concentration). The carotenoid profile of the extract revealed that the xanthophylls all-*trans*-lutein and all-*trans*-zeaxanthin were the major carotenoids extracted, owing to their polarity. Interestingly, the use of ethanol alone (75 mL/100 mL) was found to be insufficient for the extraction of carotenoids. It was anticipated that MEF was supposed to act on the cell membranes promoting their permeabilization. However, micrographic images of biomass samples revealed no visible damage caused by MEF to the cell structure, suggesting that a reversible electroporation occurred.

#### 3.1.3. High Voltage Electric Discharges (HVED)-Assisted Extraction

HVED is a cell disintegration technique based on the phenomenon of electrical breakdown of water. As illustrated in Figure 2c, during HVED treatment, the biomaterial of interest is placed in a treatment chamber with a high voltage needle electrode and a plated grounded electrode exposed to pulsed shockwaves (typically, 40–60 kV/cm, 2–5 μs) [50]. To date, the mechanisms of HVED, due to their complexity, are not well understood. However, the combination of electrical breakdown with a number of secondary phenomena (high-amplitude pressure shock waves, bubbles cavitation, creation of liquid turbulence, etc.) occurring during HVED treatment have been reported to cause cell structural damages, including cell wall disruption, which accelerates the extraction of intracellular compounds. The attempts to study the effect of HVED on the extraction of intracellular compounds from microalgae have shown that this technology is effective in achieving the extraction of water-soluble, as well as high molecular weight intracellular compounds. However, as reported in Table 1, HVED does not appear to be very effective for the extraction of pigments (e.g., chlorophylls or carotenoids), which instead requires the use of organic solvents or the application of harsher, mechanical homogenization techniques, such as US or HPH [54].

Based on the available literature data, it can be concluded that electrotechnologies (PEF, MEF and HVED) offer a considerable potential for improving the extraction of carotenoids from microalgae. However, greater research is required in order to deeply understand the mechanisms regulating the electrically induced disintegration of cell wall, plasma and organelles (chloroplast) membranes, as well as the subsequent mass transfer of the target intracellular compounds. Moreover, the efficacy of the electrotechnologies on extraction improvement requires careful optimization of process parameters, depending on the compounds of interest, as well as algal cell size, shape, and envelope structures. It is likely that the potential of electrotechnologies could be exploited by using them as a first disintegration step in a multi-stage approach. In the first stage, water-soluble compounds could be extracted; in subsequent stages, either more powerful cell homogenization techniques or “green” solvents could be applied to achieve higher extraction yields of pigments or other hydrophobic compounds.

### 3.2. Pressurized Liquid Extraction

PLE, also known as accelerated solvent extraction, has been acknowledged as a green alternative technique for the extraction of compounds from biological matrices. It was first described by Richter et al. [71] in 1996. PLE involves the extraction using liquid solvents at elevated temperature and pressure (always below their critical points), normally in the ranges of 50–200 °C and 35–200 bar, respectively [72,73]. The use of solvents at temperatures above their atmospheric boiling point reduces their viscosity and surface tension significantly and enhances solubility and mass transfer of analytes. The main advantage of using PLE is that it allows for rapid extraction and reduces solvent consumption [74,75,76,77]. PLE allows for the efficient usage of green solvents such as water and ethanol for the extraction of a wide variety of compounds by changing their dielectric constants (polarity) to values similar to those of organic solvents [72]. Although water is the most widely used polar solvent for PLE (also referred as pressurized water extraction, subcritical water extraction, superheated water extraction, pressurized hot-water extraction), alternatively, bio-ethanol [78], methanol [79,80,81,82], *n*-hexane [83], propane, dichloromethane [84], acetone [85], ethyl acetate [86], ionic liquids [87], surfactants [88] can also be applied.

To date, PLE has been extensively investigated for the recovery of commercially and industrially valuable compounds from varying plant sources (reviewed in [89,90]). Nevertheless, the use of PLE in the recovery of carotenoids from microalgae and seaweeds is relatively limited. In an early study, Denery et al. [91] demonstrated the efficiency of PLE as an alternative technology for the extraction of oxygen and light-sensitive carotenoids from two green microalgae, namely *Dunaliella salina* and *Hematococcus pluvialis*. They confirmed that PLE required a lower amount of solvent and shorter extraction times compared to traditional extraction methods. They extracted equal amount of astaxanthin, β-carotene, lutein, and total pigments from *D. salina* and *H. pluvialis* compared to the traditional method.

In a subsequent study, Herrero et al. [92] detected all-*trans*-β-carotene and its isomers along with several minor carotenoids in the extract of the microalga *Dunaliella salina* while extracting antioxidant compounds using PLE [92]. The *n*-hexane extract obtained at 160 °C for 17.5 min showed the highest levels of β-carotene isomers (25.07 mg/100 g) and total carotenoids (29.50 mg/100g). This amount was more than seven times higher than the one obtained for ethanol extracts, with the antioxidant activity displaying double the activity to that of the ethanol extract. The authors selected ethanol as the most suitable solvent for PLE for these antioxidant compounds considering the total extraction yield and reduction in environmental impact using *n*-hexane. Interestingly, the β-carotene recovery increased with the extraction temperature and the best yield was obtained at a temperature of 160 °C, indicating PLE at high temperatures was not detrimental to the extraction of carotenoids, provided a short extraction time was applied.

In another study, PLE revealed the presence of several antioxidative carotenoids in the extracts of seaweed, *Himanthalia elongata* (commonly known as sea spaghetti) and from the microalgae *Synechocystis* sp. [93]. Fucoxanthin (0.82 mg/g) and zeaxanthin (0.13 mg/g) were the major carotenoids found in the *H. elongata*, while β-carotene (2.04 mg/g) zeaxanthin (1.64 mg/g), myxoxanthophyll (0.58 mg/g) and echinenone (0.24 mg/g) were abundant in the *Synechocystis* sp. extract. Overall, the *Synechocystis* sp. ethanolic extracts obtained at 100 °C showed a higher carotenoid yield than the those obtained at different temperatures (50, 150 and 200 °C), and using other solvents (*n*-hexane and water) [93].

Similarly, PLE was found to be a suitable technique for the extraction of bioactive carotenoids from *C. vulgaris* [94]. PLE followed by HPLC-DAD analysis revealed the presence of lutein, *cis*-lutein, and β-carotene in the extract and their presence was positively correlated to their antioxidant activity. In general, PLE resulted in a greater carotenoids yield when compared to conventional maceration and UAE [94]. In a subsequent study, the PLE process was optimized and its efficiency was compared with maceration, Soxhlet extraction and UAE [95]. Ethanol at 90% was found to be a suitable solvent for the PLE of carotenoids from *C. vulgaris* when compared to acetone, *n*-hexane and water. A temperature of 116.8 °C and an extraction time of 25.1 min were found to be optimal for the extraction of β-carotene, while 48.2 °C and 34.6 min yielded an optimum quantity of lutein. At these conditions the yields of β-carotene and lutein was 0.67 and 3.70 mg/g of sample, respectively. In general, β-carotene and lutein were more effectively extracted by PLE than conventional Soxhlet extraction and maceration techniques. The efficiencies of PLE and UAE for lutein extraction were similar, however, PLE found to be less time consuming [95].

In another study, extraction of antioxidant carotenoids from the microalga *Haematococcus pluvialis* was investigated by PLE using *n*-hexane and ethanol as the extraction solvents [96]. Lutein followed by neoxanthin and β-carotene were the main carotenoids extracted from the green phase, *H. pluvialis* vegetative cells, whereas astaxanthin derivatives were observed in abundance from the red phase encysted cells formed under the stress condition. Overall, ethanol was found to be the most suitable solvent for the recovery of total carotenoids, although the *n*-hexane extract showed a higher astaxanthin content (35.1 mg/g dw) in red phase cells [96]. Plaza et al. [97] demonstrated that acetone was the most suitable solvent to extract *C. vulgaris* carotenoids (α-carotene, β-carotene, neoxanthin and violaxanthin) with respect to ethanol and water in PLE. The authors also showed that PLE provided a higher yield in carotenoid compared to UAE. In a similar study, Kim et al. [98] showed that ethanol was a suitable solvent for the extraction of fucoxanthin from the microalga *Phaeodactylum tricornutum.* PLE was performed at 100 °C for 30 min at 103 bar yielding 16.51 mg/g dw of fucoxanthin. Although the obtained yield was similar to conventional maceration methods, PLE enabled to reduce solvent use and extraction time [98].

Recently, Shang et al. attempted to optimize the PLE protocols for the efficient recovery of carotenoids, using statistical experimental designs for the extraction of fucoxanthin from the edible seaweed, *Eisenia bicyclis* [99]. Results revealed that temperature and ethanol concentration significantly influenced the extraction efficiency. Fucoxanthin was found to be relatively stable at 80 °C when extracted for 1 h, however, slight degradation was observed at 100 °C when extracted for 1 h. Optimized conditions obtained by Response Surface Methodology (RSM) revealed that at 110 °C using 90% ethanol resulted in 0.39 mg/g of fucoxanthin. In a similar study, Koo et al. [100] optimized the pressurized liquid extraction of zeaxanthin from *C. ellipsoidea* using central composite design. According to their results, the highest recovery of zeaxanthin was obtained using ethanol when compared to *n*-hexane and isopropanol. The extraction temperature showed the strongest influence on the extraction of zeaxanthin. The optimum extraction temperature and time for zeaxanthin found to be 115.4 °C and 23.3 min, respectively and the maximum yield obtained under these conditions was 4.28 mg/g. Similarly, Castro-Puyana et al. [45] attempted to optimize the extraction conditions for the recovery of carotenoids from *Neochloris oleoabundans* using PLE with food grade solvents such as ethanol and limonene. A three-level factorial design was employed to optimize the extraction conditions; at a temperature of 112 °C and 100% ethanol (0% limonene) as the extraction solvent provided optimum yields of carotenoids. Under these conditions approximately 97–98 mg/g of the extract of carotenoids were detected with lutein the major carotenoid identified in the extract. Several other secondary carotenoids including canthaxanthin, echinenone, and astaxanthin monoesters and diesters were also detected [45].

In another recent study, Taucher et al. [101] showed that use of dichloromethane as an extraction solvent in PLE yielded significantly higher levels of carotenoids from *H. pluvialis* when compared to acetone, ethanol, ethyl acetate and *n*-hexane. The extraction temperature up to 60 °C demonstrated a positive effect on the recovery of carotenoids whilst higher temperatures resulted in the degradation of carotenoids. At optimized conditions (PLE at 60 °C for 10 min, in 1 cycle, using dichloromethane), 3.69 μg/mg dw astaxanthin and 4.78 μg/mg dw total carotenoids were recovered from *H. pluvialis*. Furthermore, 1.48 μg/mg dw lutein and 1.29 μg/mg dw astaxanthin were recovered from *Chromochloris zofingiensis* and 2.08 μg/mg dw lutein was obtained from *C. sorokiniana* under these conditions. The recovery was also dependent on mechanical cell-disruption techniques used (high pressure homogenization and ball mill disruption) [101].

Overall, PLE has been demonstrated to provide an alternative for the extraction of carotenoids from microalgae and seaweeds. It is evident that ethanol and in some cases acetone [97], dichloromethane [101] are the most suitable solvents for the PLE of bioactive carotenoids rather than organic solvents such as *n*-hexane [92,93,95,96,100,102]. Ethanol noted as a “green” solvent minimizes cost and environmental impact. Although, for the extraction of carotenoids, several researchers claim that PLE reduced the solvent consumption, a comparative study is elusive. A detailed investigation including optimization data is required for the potential usage of PLE in commercial and industrial applications.

### 3.3. Supercritical Fluid Extraction (SFE)

The current literature identifies that SFE is the most extensively studied non-conventional extraction technique for the recovery of carotenoids from algae and microalgae. SFE has been considered as a sustainable “green” technology for the selective isolation of compounds. SFE uses supercritical fluids i.e., fluids at a temperature and pressure above its critical limit as the extraction solvent. Since supercritical fluids possess low viscosity and high diffusivity, they provide better solvating and transport properties than liquids. As an important advantage, the solvating power (polarity) of supercritical fluid can be adjusted by manipulating the temperature and pressure of the fluid, allowing for the selective extraction of a wide range of compounds [103]. Nowadays, many laboratories and industries are replacing conventional extraction techniques with SFE in order to minimize organic solvent consumption and increase high throughput [104]. Currently, carbon dioxide is the preferred solvent (referred as supercritical CO_2_ extraction) as it can easily attain supercritical conditions and has several advantages including low toxicity, flammability and cost, and high purity when compared to other fluids [104]. Supercritical carbon dioxide provides a nonpolar environment and its polarity can be occasionally modified by using co-solvents, such as ethanol, to extract relatively polar xanthophylls, such as lutein and astaxanthin. In some studies, ethane and ethylene were also used as SFE solvents for the extraction of carotenoids [105].

SFE technology for extraction of carotenoids has been employed from laboratory to the commercial scale. Reported applications of SFE to extract a wide range of carotenoids from microalgae and seaweeds are summarized in Table 2. In many cases, SFE has been found to be a superior technique for the extraction of heat sensitive carotenoids. A number of investigations are available to describe several SFE issues, such as the effect of temperature, pressure, co-solvents, solvent flow rate [19,106] and pretreatment, the extraction of carotenoids, selectivity [107], kinetics [29,107,108], and the modelling of extraction [26,109].

In the supercritical CO_2_ extraction of carotenoids, in general, the extraction efficiency increases with CO_2_ pressure and temperature up to a optimal level [110,111,112,113,114], nevertheless, this trend is dependent on the combined effect of pressure and temperature [19,112,115,116,117]. In some instances, high CO_2_ pressure (>400 bar) has caused lower recovery of carotenoids [108,112], and on the other hand, some researchers have observed a reduction in carotenoid yields at low CO_2_ pressure, the latter dependent on the temperature used [107,113]. Pressure has contrasting effects on the extraction yield; increasing pressure (at a constant temperature) increases the density of CO_2_, and consequently, the solvation power of the fluids, which in turn increases the solubility of the compounds and extraction yield. However, high pressure can obstruct the diffusivity of supercritical fluid into the matrix, therefore decreasing the extraction yield [112]. Similarly, an increase in temperature at a constant pressure increases the vapour pressure resulting in improved solubility of pigments. An increase in temperature results in a decrease in the fluid density, which in turn results in lower solubility of pigments. Therefore, the recovery of carotenoids is highly dependent on the complex interaction of temperature and pressure, which greatly affects density, viscosity and vapour pressure in the system. The predominance of one or other effects is responsible for the extraction efficiency [112]. For instance, Macías-Sánchez et al. [116] obtained the highest carotenoid yield from the marine microalgae *Synechococcus* sp. using supercritical CO_2_ at a temperature of 50 °C when the operating pressure was 200 and 300 bar, while the yield decreased when the pressure was increased to 400 and 500 bar. At these pressures, the maximum extraction yield was obtained when the temperature was 60 °C. The observed variation in the yield with respect to pressure and temperature was correlated to the dominating effects of density or vapour pressure at these conditions. Similar observations were made in the authors subsequent study on SFE of carotenoids from *Scenedesmus almeriensis* [112]. While studying the effects of pressure (200–600 bar ) and temperature (32–60 °C), the maximum yield of lutein was recovered at intermediate pressures, except for the extraction at 46 °C where the maximum yield was obtained at 600 bar [112]. Furthermore, a number of studies have shown similar effects of pressure and temperature on the recovery of several carotenoids [107,108,110,111,114,115,116,117,118,119,120,121].

Several researchers have used co-solvents such as ethanol [26,108,109,111,113,114,118,120,121,122,123,124,125,126,127], acetone [113], vegetable oil [26,106,128] as polarity modifiers for the efficient recovery of carotenoids such as β-carotene [113,118], astaxanthin [106,109,114,120,121,122,125,129], lutein [111,113] and zeaxanthin [118]. Since supercritical CO_2_ is non-polar, addition of a small amount of co-solvent increases the ability of supercritical CO_2_ to dissolve relatively polar carotenoids. Addition of co-solvents can cause swelling [130] of algal cells, facilitating the rapid mass transfer of analytes from the matrix [109,114]. Some co-solvents such as ethanol can enhance mass transfer by creating hydrogen bonding with analytes [114,131]. In a study on the supercritical CO_2_ extraction of lutein from *Scenedesmus* sp., ethanol was found to be the superior co-solvent compared to methanol, propanol, butanol and acetone [111]. In another study, presence of the co-solvent ethanol improved the total carotenoid recovery from the microalga *H. pluvialis* by up to 25%. Similarly, it increased the recovery of fucoxanthin from the seaweeds *U. pinnatifida* and *Sargassum muticum* by up to 90 [29] and 10 times [23], respectively. Interestingly, Krichnavaruk et al. [106] showed that vegetable oils such as soybean oil or olive oil can be used as a co-solvent to enhance astaxanthin recovery from *H. pluvialis* in supercritical CO_2_ extraction. The presence of 10% olive oil increased the recovery up to 51% at 70 °C and 400 bar, which was equivalent to that obtained using ethanol as a co-solvent. Recently, Saravana et al. [26] showed that sunflower oil as a co-solvent with supercritical-CO_2_ increased the recovery of carotenoids and fucoxanthin from the brown seaweed *Saccharina japonica* and its efficiency was greater than canola oil, soybean oil, and ethanol.

Although the use of co-solvents improves the extraction yield, in some cases, the presence of co-solvents can decrease the selectivity of the extraction [113,118]. Cardoso et al. [118] showed that although the obtained yield of β-carotene increased on using CO_2_ and 5% ethanol as a co-solvent, under these conditions zeaxanthin was co-extracted. In any case, the selectivity was also dependent on SFE parameters such as pressure and temperature [118], including extraction time [113]. Thus, the use of a co-solvent might tend to compromise product purity and should be considered.

The initial pretreatment of algae with physical or mechanical cell disintegration techniques such as crushing, sonication, ball-milling has been found to enhance the extraction efficiency in SFE [110,114,119,122]. The yield of total carotenoids obtained using SFE (with CO_2_ and ethanol co-solvent) was significantly higher when the most homogenized form of the microalga *Synechococcus* sp., was used for the extraction when compared to uncrushed cells (91.8% recovery against 58.7%, respectively) [114]. Valderrama et al. [122] found that the astaxanthin yield increased with the degree of crushing microalga *H. pluvialis* during SFE performed at 60 °C at 300 bar with CO_2_. Similar results were observed while extracting total carotenoids from *C. vulgaris* [132]. Crushing enhances the accessibility of supercritical fluids to the carotenoid bound to the cell organelles, thereby, increases extraction efficiency.

Several SFE parameters (pressure, temperature, flow rate, time, co-solvents etc.) significantly influence the extraction efficiency as well as selectivity of target compounds for extraction. Therefore, these parameters must be carefully considered and optimized for an efficient and selective recovery of target analytes. RSM could be a good statistical tool to design experiments, optimize experimental parameters and to determine the effect of these parameters on carotenoid yield. In a study Thana et al. [133] employed RSM with central composite design to investigate the effect of operating temperatures (40–80 °C), operating pressures (300–500 bar) and extraction times (1–4 h) on astaxanthin yields in supercritical CO_2_ extraction. The optimal conditions for extraction of astaxanthin were found to be at 70 °C temperature, 500 bar pressure, and 4 h time. Under these conditions, the predicted astaxanthin extraction yield was 23.04 mg/g (83.78% recovery). Recently, Saravana et al. [26] showed that ~50 °C temperature, 300 bar pressure, and 2% of sunflower oil co-solvent are the most suited conditions for the extraction of total carotenoids and fucoxanthin from the brown seaweed, *Saccharina japonica* using supercritical CO_2_. The authors attained 2.391 mg/g total carotenoids and 1.421 mg/g of fucoxanthin under these conditions. The optimum pressures and temperatures required for the extraction of carotenoids from microalgae such as *Nannochloropsis gaditana* [115,123], *Scenedesmus almeriensis* [112], *Dunaliella salina* [123]*, Chlorella vulgaris* [113]*, Scenedesmus* sp. [111]*, Synechococcus* sp. [116]*, Undaria pinnatifida* [19,20] has also been reported and shown in Table 2. Overall, SFE has been shown to be an excellent technique for the selective extraction of carotenoids from a wide range of algae and microalgae. In general, using supercritical CO_2_ alone enhances selectivity, while, efficiency can be enhanced by using co-solvents such as ethanol, however, selectivity must be compromised.

### 3.4. Subcritical Fluid Extraction

Subcritical fluid extraction is a technique, similar to SFE, where subcritical (liquefied) fluids are used as extraction solvent. Compared to SFE, only a limited number of reports are available describing subcritical fluid extraction of carotenoids from microalgae and seaweeds. Subcritical fluid extraction operates at relatively lower temperature and pressure than SFE [18,23,129,138,139]. In recent studies, subcritical CO_2_, 1,1,1,2-tetrafluoroethane and dimethyl ether (DME) have shown potential to extract carotenoids from microalgae and seaweeds [18,23,129,138,139].

Subcritical CO_2_ extraction (SCCE) uses liquid CO_2_ as the extraction solvent. It operates at relatively lower temperature (lower than critical temperature of CO_2_, 31.06 °C), and therefore is effective in extracting thermally labile compounds. In this case, the operating pressure is maintained (sometimes higher) to the critical pressure of CO_2_ (73.8 bar). Recently, Fan et al. [138] reported the extraction of lutein from *C. pyrenoidosa* using ultrasound-enhanced subcritical CO_2_ extraction (UCCE) using ethanol as the co-solvent. The authors achieved excellent recovery of lutein (124 mg/100 g) using USCCE when compared to Soxhlet extraction, subcritical water extraction and supercritical CO_2_ extraction. Table 3 compares the efficiency of UCCE with other conventional and non-conventional extraction techniques in terms of process conditions and lutein yield.

Recently, subcritical (liquefied) dimethyl ether (DME) was also used as an extraction solvent replacing CO_2_ [18,23,129]. DME below its critical temperature and pressure (critical temperature, 126.85 °C; critical pressure, 53.7 bar) can dissolve a wide range of polar and nonpolar compounds [129]. DME can enhance mass transfer by forming hydrogen bonds with extractable compounds. DME is considered a non-toxic [140] extraction solvent [129], and unlike supercritical CO_2_ extraction, raw samples can be used for the recovery of carotenoids without drying the biomass, reducing process time and cost. As an additional advantage, the liquefied DME can be evaporated as a gas under low-pressure, which is a highly effective and energy efficient method for solvent recovery [141]. Recently, Billakanti et al. [18], Goto et al. [129] and Kanda et al. [23] reported the extraction of fucoxanthin from *U. pinnatifida* using subcritical DME. At 25 °C, 5.9 bar pressure and 0.72 h extraction time, the amount of fucoxanthin recovered was approximately 390 μg/g dw [23,129], which was significantly higher than that attained by conventional Soxhlet extraction using ethanol (50 μg/g dw) [23]. However, the yield was lower than that obtained in supercritical CO_2_ extraction [23]. The recovery of fucoxanthin using conventional extraction, subcritical DME extraction and supercritical CO_2_ is compared in Table 4 [adapted from ref. [23]]. In a study, Billakanti et al. [18] found that enzyme pretreatment prior to subcritical DME extraction has no significant positive effects on the recovery of fucoxanthin from wet or dry *U. pinnatifida* biomass, however, the addition of ethanol as a co-solvent slightly enhanced the relative recovery from the wet biomass [18].

In a recent subcritical fluid extraction study, carotenoids of marine seaweed *Laminaria japonica* were extracted using ethanol-modified subcritical 1,1,1,2-tetrafluoroethane (R134a) [142]. Response surface methodology (RSM) combined with a Box–Behnken design was applied to investigate the effects of pressure (50–170 bar), temperature (30–50 °C) and the amount of co-solvent (2%–6% R134a, *w*/*w*) on the recovery of carotenoids. An extraction temperature of 51 °C, extraction pressure 170 bar and a co-solvent amount of 4.73% yielded optimum quantity of carotenoids (0.233 g/kg), however, the yield was lower than that obtained using UAE with methanol solvent (0.336 g/kg) [142].

### 3.5. Microwave-Assisted Extraction

Microwaves are non-ionizing electromagnetic radiations with a frequency ranging from 300 MHz to 300 GHz. Microwave radiation can transfer heat to the system by means of dipole rotation of molecules and ionic conduction in the medium. This principle has been the basis for the development of microwave-assisted extraction (MAE), where the extraction is facilitated by microwave radiation that transfers heat in the extraction medium and aids in the dissolution and mass transfer of analytes. Heat transfer resulted by microwave irradiation can also cause evaporation of moisture inside the cell, developing significant pressure inside the biological matrix. This pressure change can rupture cell membranes and increase the cell porosity, which in turn accelerates the penetration of solvent and the release of intracellular compounds. Microwave radiation can also cause the disruption of hydrogen bonds and migration of dissolved ions, which further enhances the extraction of analytes (for an extensive explanation on theory and principles, see [143]). MAE can be performed in open or closed reaction vessels. Open vessels are used for low temperature extraction at atmospheric pressure whereas closed vessel systems are used for high temperature extractions.

Recently, several researchers have shown that MAE has the potential for the recovery of carotenoids from microalgae and seaweeds. Existing reports suggest that the efficiency of MAE is mainly dependent on the extraction condition and algal cell structures. In some cases, researchers have observed the selective degradation of carotenoids such as astaxanthin during intense microwave treatments, but not the subsequent degradation of other carotenoids such as fucoxanthin. Mild microwave treatment is sufficient to extract compounds from algae, however, species with complex exopolysaccharide envelopes require slightly intense microwave treatment. In a study, fucoxanthin was extracted from a frustulated diatom, *Cylindrotheca closterium* in acetone using MAE technique by Pasquet et al. [144]. MAE at 50 W resulted in the total extraction of fucoxanthin in 3–5 min with an extraction yield of 4.24 μg/mg. The yield obtained by MAE was comparable to that obtained by conventional cold and hot soaking extractions performed for 60 min (4.68 and 5.23 μg/mg, respectively), nevertheless, MAE significantly reduced the extraction time. MAE assisted the disruption of frustule structure associated with diatoms helping in the rapid extraction of analytes. In their study, increasing microwave power and irradiation times did not show any effect on the extraction yield, indicating a higher stability of fucoxanthin under microwave radiation. In the same study, authors did not observe a significant improvement in the extraction yield for β-carotene isolated from *Dunaliella tertiolecta* when they compared MAE with other conventional methods. This was mainly due to a simple cell-wall structure associated with *D. tertiolecta*, which may not require additional energy for cell disruption and mass transfer.

MAE has also been successfully applied for the extraction of fucoxanthin from seaweeds. In one study Xiao et al. [21] showed the optimized process conditions for MAE of fucoxanthin from edible seaweeds and brown algae. *U. pinnatifida* was used as model matrix for the optimization process, and the solvent/sample ratio, irradiation time was significant on the recovery of fucoxanthin, whereas, the microwave power had insignificant influence. The use of ethanol and acetone as extraction solvent resulted in similar extraction yields, whilst a 50% *n*-hexane in ethanol caused lower recovery of fucoxanthin. Applying ethanol as the extraction solvent, with a solvent to sample ratio of 15:1 mL/g, an extraction temperature of 60 °C, a time of 10 min and microwave power of 300 W resulted in the optimum recovery of fucoxanthin. Under these conditions, the maximal yield of fucoxanthin from fresh *L. japonica*, dry *U. pinnatifida*, and dry *S. fusiforme* was 5.13, 109.3, and 2.12 mg/100 g, respectively. Based on the studies of Pasquet et al. [144] and Xiao et al. [21] it can be concluded that microwave energy optimal level does not affect the stability of fucoxanthin, indicating fucoxanthin is relatively stable carotenoid under microwave irradiation.

MAE offers great potential for the extraction of astaxanthin from microalgae. In one study, a closed system MAE resulted in the highest astaxanthin recovery from marine alga *H. pluvialis* in a shorter duration (5 min) when compared to conventional solvent extractions and ultrasound assisted extractions (UAE), at a time of 60 min for the recovery of astaxanthin [145]. Acetone was found to be a suitable solvent for the recovery of astaxanthin when compared to methanol, ethanol and acetonitrile. MAE at a temperature of 75 °C resulted in 74% recovery of astaxanthin. A temperature above 75 °C in the microwave system caused a rapid loss in the recovery of astaxanthin [145]. Most of the carotenoids are temperature sensitive due to their structure and chemical bonding and can undergo isomerization and/or degradation at elevated temperature [146,147]. Therefore, it is important to optimize the process parameters to recover these molecules in an acceptable yield and purity. Optimizing this process also aids in the reduction of solvent and overall energy consumptions. In this approach, Zhao et al. [148] attempted to optimize MAE of astaxanthin from *H. pluvialis* in ethanol and ethyl acetate (2:1, *v*/*v*) medium using RSM [148]. The authors found that the extraction parameters such as microwave power, extraction time, solvent volume, the number of extraction, and their interaction effects had significant influence on the recovery of astaxanthin. A microwave power of 141 W, extraction time of 83 s, solvent volume of 9.8 mL and four consecutive extractions was found to be optimum for the recovery of astaxanthin provided yields of about 5.94 μg/mg dw. The response surface plots showed that an increase in microwave power beyond 141 W decreased the recovery of astaxanthin. Higher microwave power can lead to increase in the temperature of the extraction medium, which can disrupt the structure of astaxanthin, leading to its lower recovery [148].

Recently, Esquivel-Hernández et al. [126] showed that MAE is an excellent technique for the recovery of total carotenoids from microalgae/cyanobacteria *Arthrospira platensis* (Spirulina). MAE extraction performed using a mixture of methanol/ethyl acetate/light petroleum (1:1:1 *v*/*v*) at 400 W power, 50 °C temperature and 1 bar pressure, for 15 min yielded 629 μg/g of total carotenoids, which was significantly higher than the yield obtained by SFE (283 μg/g) (ref). In general, it can be concluded that MAE is a promising technology for the rapid extraction of carotenoids. However, in this case microwave power and extraction temperature must be accurately adjusted; as it could lead to the subsequent degradation of selected, valuable carotenoids.

### 3.6. Ultrasound-Assisted Extraction

Ultrasound is composed by sound pressure waves ranging from 20 kHz to 10 MHz with intensities greater than 1 W/cm^2^ which can be disruptive to matter, depending on the frequency utilized [149]. Most applications in extraction have dealt with low frequency ultrasound, defined between 18 and 200 kHz and recent literature defined the application of high frequency ultrasound standing waves between 400 kHz and 2 MHz to enhance separation [150].

The propagation of ultrasonic waves through liquid medium results in alternating compression and rarefaction cycles. During these rarefaction cycles, small bubbles filled with vapors are created, and these bubbles are able to grow to a certain size and shrink periodically. The formation of small bubbles in a liquid is defined as cavitation. Low frequency ultrasound produces large bubbles and bubble size decreases with frequency [151]. Bubbles formed at lower frequencies (between 18 and 200 kHz) typically attain a critical diameter up to several microns and collapse during the compression cycle, releasing large amounts of heat and shockwaves, creating localized temperatures around 5000 K and pressure jets from strong bubble implosions due to unstable cavitation [149]. Conversely, sound waves produced at frequencies in the megasonic range (>1MHz) produce more stable cavitation, producing tiny bubbles that open and close, creating localized microstreaming effects. Cavitation in general enhances diffusion through cell membranes; furthermore, the high temperature and pressure generated due to low frequency unstable cavitation can also destroy cell structures releasing intracellular components into the medium [152]. Therefore, low frequency ultrasound, also known as ultrasound-assisted extraction (UAE), has been more largely explored for the extraction of components from biological matter (Table 5). UAE is an alternative extraction technique that can be performed using four types of equipment, (i) ultrasonic bath; (ii) ultrasonic probe; (iii) ultrasound plates; and (iv) tubular devices populated with small transducer ceramics. In UAE, a number of parameters such as ultrasonic power, frequency, intensity, shape and size of the ultrasonic reactor, solvent type, temperature, presence of dissolved gases and an external pressure greatly influences the extraction efficiency (recently reviewed by [153]). Extraction temperatures can be controlled during UAE using heat-exchange systems, which are helpful in extracting thermally labile compounds in particular carotenoids.

In recent years, UAE has been employed to extract fucoxanthin, lutein, β-carotene, and astaxanthin from microalgae and seaweeds (Table 5). In a study Macías-Sánchez et al. [117] investigated the efficiency of UAE for the recovery of total carotenoids from the microalga *D. salina*. UAE was performed using lyophilized samples using methanol and *N*,*N*-dimethylformamide (DMF) as extraction solvents. According to their results, UAE performed using DMF recovered up to 27.7 μg/mg dw carotenoids and was significantly higher than the yield obtained by SFE (up to 14.92 μg/mg dw). On the other hand, the UAE had lower selectivity for carotenoids when compared to SFE [117].

In another study, Pasquet et al. [144] compared the extraction efficiency of UAE, MAE and conventional, cold and hot soaking methods for the extraction of fucoxanthin from *Cylindrotheca closterium* and β-carotene from *Dunaliella tertiolecta*. Although UAE performed at a power level of 4.3–12.2 W which appeared to be a rapid extraction technique compared to conventional soaking extractions and did not improve pigment yields at tested conditions. Similar results were observed in a subsequent study performed using the microalga, *Phaeodactylum tricornutum* [98], where the fucoxanthin yield attained through UAE was 15.96 mg/g dw was similar to conventional Soxhlet extraction and maceration; however, UAE reduced the extraction time significantly. Controlling process parameters or adjusting the power level could enhance the recovery of these pigments from these microalgae.

UAE was found to be a suitable technique also for the of extraction of violaxanthin, neoxanthin, β-carotene and lutein from *C. vulgaris* [97]. Superior extraction efficiency was obtained with acetone when compared to ethanol or *n*-hexane. However, UAE resulted in significantly lower carotenoids when compared to PLE [97]. Similar observations were also deduced by Cha et al. [95], who reported that a lower recovery of β-carotene was obtained in UAE when compared to PLE. On the other hand, lutein was recovered in similar levels both in UAE and PLE (3.83 and 3.78 mg/g, respectively [95]. Optimization of the UAE process parameters is crucial to increase pigment recovery. For example, Dey et al. [158] studied the effects of various UAE parameters such as extraction time, solvents, solid to solvent ratio, temperature, intensity, probe immersion length, duty cycles and pretreatment on the extraction of β-carotene from *Spirulina platensis.* Authors found that *n*-heptane was a better solvent than *n*-hexane and diethyl ether for ultrasound-assisted recovery of β-carotene. The extraction rate increased up to 4 min, and then slowed down, reaching saturation at 8 min. While investigating the effect of electrical acoustic intensity (in the range of 64–210 W/cm^2^), the extraction yield increased up to 167 W/cm^2^, then decreased at 185 W/cm^2^ and increased again at 210 W/cm^2^. The higher extraction yield observed up to 167 W/cm^2^ was attributed to the increase in cavitation, whereas the lower yield at 185 W/cm^2^ was ascribed to the formation of excessive bubbles due to higher acoustic intensity, which hindered the propagation of waves resulting in lower recovery. The authors suspected that a higher yield obtained from 185 to 210 W/cm^2^ was due to the positive influence of thermal effects generated at higher electrical acoustic intensities [158]. Since the authors have performed experiments utilizing a one-factor-at-a-time approach, the effect of uncontrolled parameters or interaction effects of controlled parameters on the extraction process could be a plausible reason for this ambiguous observation. On a comprehensive level, 1.5 g sample (2 min pre-soaked in methanol) in 50 mL *n*-heptane at 30 °C temperature, 167 W/cm^2^ electrical acoustic intensity and 61.5% duty cycle for 8 min with probe dip length of 0.5 cm resulted in the optimum recovery of β-carotene (up to 47%) [158]. Interestingly, a pretreatment of samples in methanol for 2 min dramatically enhanced the extraction yield of 12 times [158].

Similarly, Deenu et al. [156] optimized UAE and UAE combined with enzymatic pretreatment for the recovery of lutein from the green microalga *C. vulgaris* using RSM. UAE was performed at a frequency of 35 kHz and intensity of 56.58 W/cm^2^ using 90% ethanol as an extraction solvent. Ultrasonic treatment at 37.7 °C with solvent to solid ratio of 31 mL/g for 300 min resulted in an optimal lutein yield of 3.16 mg/g (wet basis). Enzymatic pretreatment for 2 h using viscozyme [1.23% (*v*/*w*)] reduced the extraction time from 300 min to 162 min with a slight increase in lutein yield (3.36 mg/g). Enzymatic pretreatment using cell-wall degrading enzymes aid cell disruption techniques [159], facilitated the recovery of analytes in subsequent UAE.

A recent optimization study by Zou et al. [155] revealed that ultrasound irradiation power of 200 W, frequency 40 kHz, a solvent composition 48.0% ethanol in ethyl acetate, liquid-to-solid ratio 20:1 (mL/g), extraction time 16.0 min and a temperature 41.1 °C resulted in the optimum recovery of astaxanthin (27.58 mg/g) from marine the microalgae, *Haematococcus pluvialis*. In this case, a higher extraction temperature and longer irradiation time also resulted in the degradation of astaxanthin with a similar result observed by Ruen-ngam et al. [145]. In another study, the effect of the ultrasonic treatment on microalgal cell disruption was investigated by evaluating the release of intracellular carotenoids from the green microalga, *Chlamydomonas reinhardtii* [154]. Although this research work was not aimed at extracting carotenoids, cells were sonicated under cold conditions for 10 or 30 s at amplitudes of 160, 128, 96, 64, and 10 μm and energy levels of ≥800 J/10 mL, with results showing 0.3 μg carotenoids/mg of cells.

Recently, UAE was successfully employed to the extraction of carotenoids from the microalgae, *Phormidium autumnale* cultivated from agro-industrial wastes [160]. The carotenoids were extracted from dried microalgae in acetone for 20 min at 20 °C using an ultrasonic probe instrument. The amplitude and frequency applied for the extraction was ~61 μm and 20 kHz, respectively and the ultrasound probe depth was 25 mm inside the sample. HPLC-PDA-MS/MS analysis identified the presence of twenty carotenoids. The all-*trans*-β-carotene (70.22 μg/g), all-*trans*-zeaxanthin (26.25 μg/g), all-*trans*-utein (21.92 μg/g), all-*trans*-echinenone (19.87 μg/g), *cis*-echinenone (15.70 μg/g) and 9-*cis*-β-carotene (15.70 μg/g) was found to be the major carotenoids present in *P. autumnale* [160]. Minor carotenoids identified (amount >6 μg/g) included all-*trans*-neoxanthin, 9-*cis*-neoxanthin, 9-*cis*-violaxanthin, 13-*cis*-lutein, 13′-*cis*-lutein, 13-*cis*-zeaxanthin, 9-*cis*-lutein, 9-*cis*-zeaxanthin, all-*trans*-canthaxanthin, *cis*-carotenoid, all-*trans*-myxoxantophyll, all-*trans*-zeinoxanthin and α-carotene. The total carotenoid content extracted was 183.03 μg/g [160].

Yamamoto et al. [157] have compared the efficiency for disruption of *Dunaliella salina*, a high producer of carotenoids, using both low and high frequencies. They have shown that even though low frequencies were most effective, higher frequencies between 580 and 1146 kHz also had high impact on cell disruption. High frequency transducer plates are now used in industrial applications for oil separation by promotion of droplet coalescence and through microstreaming “cleaning” mechanisms in vegetable matter and these systems could be amenable for integration of carotenoid production processes [150,161]. Standing wave high frequency (also known as megasonic) units have also shown the possibility for algal agglomeration and pre-separation during harvest, which may facilitate extraction process pretreatments [162].

UAE can be used as a pretreatment step or complementary technique with other extraction methods. In a study Fan et al. [138] showed that UAE combined with subcritical CO_2_ extraction is an efficient technique for the extraction of lutein from the microalga, Chlorella pyrenoidosa [138]. The combination of UAE and enzyme-assisted extraction for the extraction of lutein was previously described by Deenu et al. [156]. Recently, Parniakov et al. [63] found that the sonication pretreatment (60–600 s at power of 400 W and frequency of 24 kHz), followed by pH assisted extraction was efficient in the extraction of carotenoids from *Nannochloropsis* sp. However, ultrasonic pretreatment resulted in lower selectivity and required supplementary purification of the final products. Additional, sonication consumed greater power (≈250 kJ/kg, 200 W, 600 s) when compared to PEF (≈100 kJ/kg, 20 kV/cm, 6 ms).

### 3.7. High Pressure Homogenization (HPH)

**Figure 3:**
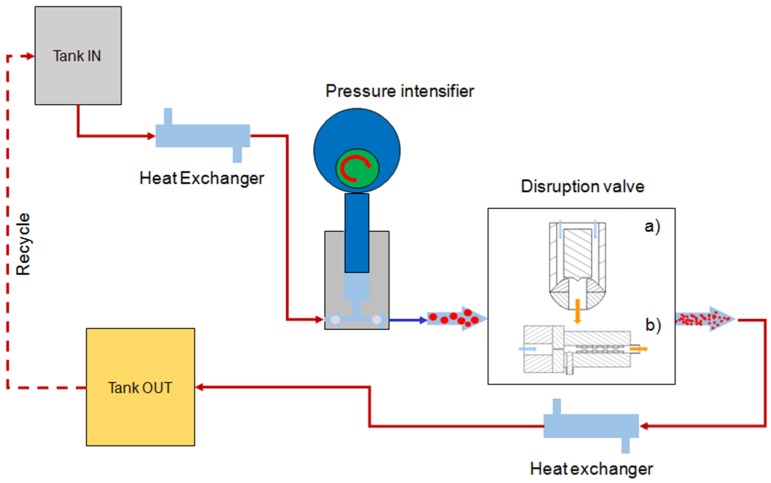
Schematics of a high pressure homogenization system, equipped with a (**a**) piston; or (**b**) orifice disruption valve.

A physical or mechanical pretreatment prior to extraction can be exploited to disrupt the cell wall of microalgae, promoting the recovery of carotenoids. In fact, to achieve high product yields, efficient cell disruption and extraction steps are required [101]. High pressure homogenization (HPH) is a wet milling process, where particle or cell disruption is achieved by applying high intensity fluid-mechanical stresses, as a consequence of the flow of the process fluid under high pressures (50–400 MPa) through a specifically designed homogenization valve chamber [163]. Schematic of a HPH system is given in Figure 3. Despite each manufacturer offers specific proprietary disruption valve chambers, the designs can be broadly classified as piston valves, where the valve gap is adjusted to set the operating pressure, and orifice valves, with a fixed opening, and operating pressure being adjusted by controlling the flow rate at the pressure intensifier [164]. In comparison with other physical comminution processes, such as ball or colloid milling, and ultrasounds, it offers significant advantages in terms of ease of operation, industrial scalability, reproducibility and high throughput [164,165,166].

HPH is a promising technique, particularly applied to micro- and macroalgae, as it is effective with respect to aqueous and/or fresh samples up to 25% *w*/*w* solids [167], omitting the energy intensive drying steps, and can be easily scaled up production purposes [168,169,170]. In HPH, the extraction process is facilitated by the mechanical disruption of the cell wall and cell membranes, enabling the non-selective release of the intracellular compounds. Several factors associated with HPH contribute to cell wall disruption, which includes the development of high pressure gradients, turbulence, cavitation, collision with hard surfaces, viscous and high pressure shear, pressure drop, as well as temperature increases due to the inherent heating associated to the rapid reduction in pressure [163,165,171,172]. In particular, while some of them depend on selected operating conditions, others are affected by suspension properties (density and viscosity) and by the feed concentration. Previously reported data have shown no significant impact on process performance for feed concentrations up to 9% *w*/*w* for *Desmodesmus* sp. [173] and up to 25% *w*/*w* for *Nannochloropsis* sp. [167].

Recent studies have suggested that the HPH systems exhibit higher microalgal disruption efficiencies than other methods, such as those based on electrotechnologies (PEF, and HVED) [54], ultrasounds (US) [54,101,174,175], microwaves (MW) [175], ball milling, colloidal milling, freeze drying or thawing [101]. Moreover, when used as a pretreatment to enhance extraction, HPH resulted in significantly higher yields of intracellular compounds, such as lipids [174,175,176,177,178] or carotenoids [101,173]. During HPH, due to the balance establishing between cell wall resistance and tearing forces transmitted to cell walls by the fluid-mechanical stresses generated in the fluid upon its passage through the homogenization valve, a certain variability in the degree of cell disruption efficiency can be observed. Previous comparative studies of different valve geometries on HPH-induced cell disruption showed that piston valves in general ensure a higher homogenization efficiency than orifice ones [165]. Moreover, available reports suggest that pressure is a more important variable than the number of passes [54,101,167,170,174,175,176,177,178,179,180] (which translates in higher investment costs but lower operating costs) and that, at least up to 25% *w*/*w*, biomass concentration does not significantly affects HPH performance, with significant advantages in terms of process intensification.

Although, the HPH technique has been successfully applied to the extraction of lipids, pigments, proteins, sugars etc. from algae and microalgae [174,181,182,183], to date, only a limited number of research has investigated the efficacy of HPH on the recovery and improvement of carotenoids from algae and microalgae. In an early study, HPH was used (700 bar, 1 pass) for commercial large scale production of astaxanthin from *Haematococcus pluvialis* [170]. In another study, HPH (1500 bar, 1–10 passes, 150–1500 kJ/kg) was found to be effective to extract carotenoids from the microalgae, *Nannochloropsis* sp. [54]. Recently, Taucher et al. [101] found that HPH (1000 bar, 3 passes) pretreatment resulted in the highest total carotenoid extraction yield [4.21 μg/mg (dry weight)] from *H. pluvialis* when compared to other cell disruption methods used such as ball mill [3.56 μg/mg (dry weight)], Ultra Turrax [3.42 μg/mg (dry weight)] and repeated freeze-and-thaw-cycles [0.02 μg/mg (dry weight)]. The HPH pretreatment also assisted in enhanced recovery of lutein and astaxanthin from *C. zofingensis* [101].

Although HPH has shown to be a useful technique for cell disruption and subsequent recovery of analytes, its main disadvantage lies in the non-selective extraction of compounds. Generally, HPH targets desired products along with high amounts of cell debris/other intracellular matrices, which complicates downstream separation and purification processes. Moreover, the usage of very high pressures in the process can result in increases in temperatures (in the range of 15–20 °C/1000 bar), which can cause negative effects on the recovery of thermally labile compounds, such as carotenoids, if adequate and rapid cooling is not applied [164]. Nevertheless, the use of lower pressures and multi-step treatments, together with process optimization, could be useful in selective extraction of intracellular components [183]. To date and to our knowledge, no results have been reported about the use of HPH in the treatment of seaweeds, whose processing would require preliminary comminution steps to transform them into a slurry which can be pumped through the HPH systems (average size <500 μm).

## 4. Conclusions and Future Perspectives

It is evident that microalgae and seaweeds are sources of commercially and industrially valuable, health-promoting and biologically active carotenoids. This comprehensive review has focused on a number of emerging and innovative alternative extraction technologies, including PEF-, MEF-, and HVED-assisted extractions, PLE, SFE, subcritical fluid extraction, MAE, UAE, and HPH, used for the extraction of carotenoids from microalgae and seaweeds. Growing evidence suggest that these non-conventional techniques offer superior efficiency, selectivity, and a reduction in treatment time or solvent consumption. Nevertheless, available reports clearly indicate that the algal cell structure has a marked influence on extraction efficiency.

In general, MAE and UAE are suitable for rapid extraction, whilst PLE reduces solvent consumption. However, temperatures associated with these techniques can cause degradation of thermolabile carotenoids. On the other hand, PEF was found to be an excellent, non-thermal process for the recovery of thermally labile carotenoids. As previously mentioned, PEF was also found to be inefficient when the alga of interest had a complex cell-wall structure. SFE using CO_2_ as a solvent was documented to be a superior “green” technique for the selective extraction of carotenoids, and the extraction efficiency was drastically improved by using co-solvents such as ethanol; however, the disadvantage was that it compromised the selectivity of carotenoids. In addition to this, SFE requires careful optimization of various factors to improve extraction efficiency often requiring dried samples, which may increase time and the cost of extraction. HPH was found to be one of the most effective techniques for completely unlocking algal, intracellular compounds, enabling the rapid extraction of carotenoids; however, its lack of selectivity has been documented as a major limitation, suggesting its use only in combination with other technologies, in the form of a biorefinery concept. More recently, subcritical fluid extraction and MEF-assisted extraction has also shown a potential to extract carotenoids.

Finally, it is evident that existing and emerging, non-conventional techniques could be a sustainable alternative in comparison to traditional extraction methods. Future developments should be directed towards overcoming the limitations associated with these techniques, the hyphenating and optimizing processes for improving yield and selectivity, and reducing the multitude of instrumentation, energy costs, and scaling-up of these processes from both commercial and industrial applications. The energy and cost efficiency of these emerging technologies, particularly electrotechnologies, are still under debate, as most of them have been tested only at lab or pilot scale, and are still far from commercial readiness. A detailed scientific investigation of these technologies at the industrial scale is required to understand their commercial viability. These developments could provide an innovative avenue to increase the production and target of selected carotenoids to use as nutraceuticals, pharmaceuticals, cosmeceuticals, food, and feed supplements.

## Figures and Tables

**Table 1:** Summary of reported applications of electrotechnology (PEF, MEF, and HVED)-assisted extraction of carotenoids from microalgae. PEF: pulsed electric field; MEF: moderate electric field; HVED: high-voltage electric discharges.

Microalgae	Biomass Concentration	Electrical Treatment	Extraction Conditions	Carotenoid Yield	Notes	Reference
MEF						
*Heterochlorella luteoviridis*	4 g dry biomass/100 mL 25% ethanol solution	0–180 V, 60 Hz, 10 min, <35 °C	25%–75% ethanol, 50 min, 30 °C	Total carotenoids 1.21 mg/g dw	MEF induced a reversible electroporation improving the extraction efficiency. Xanthophylls all-*trans*-lutein and all-*trans*-zeaxanthin were the major carotenoids extracted.	[64]
PEF						
*Chlorella vulgaris*	~3% dw	15 kV/cm, 100 kJ/kg	N/A ^a^	Total carotenoids 525% recovery compared with the conventional ball milling homogenization process	Antioxidant activity of the extract was increased by almost 100%.	[53]
*Chlorella vulgaris*	10^9^ CFU/mL in McIlvaine buffer (pH 7)	10–25 kV/cm 0.6–93 kJ/L of culture	96% ethanol, 20 °C, 0–1 h	Total carotenoids: ~0.82 mg/g dw and 1.04 mg/g dw after, respectively, 0 h and 1 h of incubation after PEF treatment	Extraction yield significantly increased after 1 h of the application of PEF, likely caused by the plasmolysis of the chloroplast during the incubation time.	[60]
*Chlorella vulgaris*	2 × 10^8^ CFU/mL in McIlvaine buffer (pH 7)	Millisecond range: 1–40 ms pulses, 3.5–5 kV/cm 9–150 kJ/L of culture Microsecond range: 3 μs pulses 10–25 kV/cm 1.5–93 kJ/L	96% ethanol 20 °C, 0–1 h	Total carotenoids: ~1.06 mg/L after 0 h and 1 h of incubation in the ms range; 1.09 mg/L and 1.58 mg/L after, respectively, 0 h and 1 h of incubation in the μs range	PEF in the ms range at a lower electric field strength created irreversible alterations, while in the μs range the defects were a dynamic structure along the post-pulse time. Higher energy efficiency of treatment in the μs range than in the ms range.	[61]
*Chlorella vulgaris*	10^9^ CFU/mL in McIlvaine buffer (pH 7)	10–40 °C 10–25 kV/cm 1.5–93 kJ/L of culture	96% ethanol, 20 °C, 0–1 h	Lutein up to 0.753 mg/g dw	Increasing temperature increased the sensitivity of microalgae cells to irreversible electroporation, and decreased the total specific energy required to obtain a given extraction yield. PEF treatment did not cause pigment degradation.	[56]
*Spirulina platensis*	~3% dw	15 kV/cm, 100 kJ/kg	N/A ^a^	Total carotenoids 150% recovery compared with the conventional ball milling homogenization process	Antioxidant activity of the extract was increased by almost 100%	[53]
*Nannochloropsis* sp.	1% (*w*/*w*) in distilled water	20 kV/cm, 1–4 ms, 13.3–53.1 kJ/kg	N/A ^b^	N/A ^c^	PEF allowed selective extraction of water-soluble ionic components and water-soluble proteins, but was ineffective for extraction of pigments.	[54]
*Nannochloropsis* sp.	1% (*w*/*w*) in distilled water	20 kV/cm, 0.01–6 ms, 13.3–53.1 kJ/kg	Distilled water, up to 3 h, 50 °C, pH = 8.5–11	Total carotenoids: ~0.04 mg/g dw after PEF (pH 8.5); ~0.2 mg/g dw after PEF (pH 8.5) + extraction at pH 11	Extraction efficiency after PEF (pH 8.5) was comparable with that of the aqueous extraction at pH 11. PEF (pH 8.5) treatment was more efficient than PEF (pH 11) treatment. Supplementary extraction at pH = 11 allowed a noticeable increase of the concentrations yield. PEF extracts showed high purity.	[63]
*Nannochloropsis* sp.	1% (*w*/*w*) in distilled water	20 kV/cm, 0.01–6 ms, 13.3–53.1 kJ/kg	Aqueous DMSO, ethanol solutions: 0%, 30%, 50%, and 100%; 20 °C; 240 min	K_PEF_^d^ ≈3.0 at 50% DMSO K_PEF_^d^ ≈ 2.4 at 30% EtOH	High levels of extracted proteins at the first step with water, and noticeable enhancement of extraction of pigments at the second step with binary mixtures. The two-stage PEF-assisted procedure allowed effective extraction using less concentrated mixtures of organic solvents with water.	[62]
HVED						
*Nannochloropsis* sp.	1% (*w*/*w*) in distilled water	40 kV/cm, 1–4 ms, 13.3–53.1 kJ/kg	N/A ^a^	N/A ^c^	Noticeably agglomeration of microalgae cells in the HVED-treated suspensions. Higher pigment recovery than PEF, but less than UAE and HPH.	[54]

^a^ N/A: not available; ^b^ Extract analyzed immediately after electrical treatment; ^c^ Results provided as UV absorption spectra and absorption peaks at 415 nm; ^d^ PEF efficiency coefficient defined as the ratio of concentration values of the extracts obtained for two-stage (PEF/water extraction + extraction with binary mixture) and one-stage (extraction with binary mixture); dw—dry weight.

**Table 2:** Applications of supercritical fluid extraction (SFE) for recovery of carotenoids from algae and seaweeds.

Microalga/Seaweed	Extraction Condition					Carotenoid Yield	Notes	Reference
	Solvent ^a^	Pretreatment	P ^b^ (bar)	T ^c^ (°C)	T ^d^ (h)			
Microalgae								
*Haematococcus pluvialis*	CO_2_ and 9.4% ethanol	Crushing and then grinding in dry ice	300	60	–	Astaxanthin >97% recovery	Co-solvent enhanced the recovery slightly	[122]
*Synechococcus* sp.	CO_2_	Freeze drying	500	60	4	Total carotenoids 2.76 mg/g dw ^e^	Optimal extraction conditions for β-carotene was 50 °C, 358 bar; for β-cryptoxanthin was 59 °C, 454 bar; and for zeaxanthin was 60 °C, 500 bar.	[134]
*Haematococcus pluvialis*	CO_2_	Drying (powder form)	70	500	4	Astaxanthin 23.04 mg/g dw ^e^	Pressure, extraction time, and the interaction between temperature and pressure had significant effect on astaxanthin yield.	[133]
*Dunaliella salina*	CO_2_	Homogenization	60	400	3	Total carotenoids 12.17 mg/g algae dw	SFE was more selective than the UAE.	[117]
*Chlorella vulgaris*	CO_2_ and 7.5% ethanol	–	80	500	3	Lutein ~≥1.8 mg/g algae; β-carotene ~≥0.2 mg/g	Supercritical CO_2_ has high selectivity for lutein extraction, however the yield was lower than Soxhlet extraction; ethanol was better co-solvent than acetone.	[113]
*Chlorococcum littorale*	CO_2_ and 10 mol % ethanol	Freeze drying	60	300	1–3	Total carotenoids 0.094%–0.21% dw	Co-solvent enhanced the recovery slightly.	[127]
*Scenedesmus* sp.	CO_2_ and ethanol (30 mol %)	Freeze drying and milling	70	400	1	Lutein 2.210 mg/g algae ^e^	Higher temperature lead to increased impurity.	[111]
*Nannochloropsis gaditana*	CO_2_	Freeze drying (powder form)	60	400	3	Total carotenoids 0.343 mg/g algae dw ^e^	Higher temperature lead to degradation.	[115]
*Haematococcus pluvialis*	CO_2_ and 5% ethanol	–	70	550	4	Astaxanthin 77.9% recovery with respect to 34.3 mg/g dw total content found in the sample using Soxhlet extraction	Astaxanthin yield increased with increasing cosolvent concentration up to 5% (*v*/*v*) ethanol.	[120]
*Haematococcus pluvialis*	CO_2_ and 10% ethanol	Freeze drying and ball milling	60	300	–	Carotenoid recovery 92%; esterified astaxanthin ~75%; lutein >90%; astaxanthin >90%; β-carotene >90%; and canthaxanthin ~85%	Crushing improved the recovery significantly.	[114]
*Chlorella vulgaris*	CO_2_ and 5% ethanol	Crushing	40	300	–	Total carotenoids up to 0.299%	Crushing increased pigments recovery.	[132]
*Dunaliella salina*	CO_2_	Freeze drying	9.8	443	1.6	Total carotenoids 6.72% (predicted)	Higher yields were obtained at high pressures and low temperatures.	[135]
*Nannochloropsis* sp.	CO_2_ and 20% ethanol	Ball milling	40	300	>1	–	Co-solvent increased the yield.	[136]
*Scenedesmus almeriensis*	CO_2_	Freeze drying (powder form)	60	400	5	Lutein 0.0466 mg/g dw ^e^ β-carotene 1.5 mg/g dw ^e^	Recovery was lower compared with conventional acetone extraction.	[112]
*Synechococcus* sp.	CO_2_	Freeze drying	50	300	3	Total carotenoids 1.511 mg/g algae dw ^e^	The highest carotenoids/chlorophylls selectivity was obtained at 200 bar and 60 °C.	[116]
*Nannochloropsis oculata*	CO_2_ and 16.7 wt % ethanol	–	50	350	–	Total carotenoids 7.61 mg/g dw	Anti-solvent precipitation of carotenoids allowed pure Zeaxanthin.	[124]
*Nannochloropsis oculata*	CO_2_ and ethanol	Grinding and freeze drying	50	350	–	Zeaxanthin 13.17 mg/g	Ethanol as a co solvent increased the yield, and was efficient than dichloromethane, toluene and soybean oil	[128]
*Monoraphidium* sp.	CO_2_ and ethanol	Freeze drying	60	200	1	Astaxanthin 2.02 mg/g dw	Ethanol as a co-solvent improved astaxanthin yield.	[125]
*Chlorella vulgaris*	CO_2_ and ethanol	Pretreatment process using alcohol as elution solvent	40	400	0.75	Lutein 1.78% recovery based on 7.9 mg/g obtained in Soxhlet extraction	Ethanol as an elution solvent removed chlorophyll *a*, *b* and β-carotene and improved selectivity of lutein	[107]
*Haematococcus pluvialis*	CO_2_ and 10% olive oil	Drying	70	400	5	Asthaxanthin 51% recovery	Olive oil co-solvent lead to a recovery comparable to ethanol as a co-solvent.	[106]
*Nannochloropsis gaditana*	CO_2_ and 5% ethanol	Freeze drying	40–60	100–500	3	Carotenoid yield up to 0.3%	Extraction kinetics was studies.	[108]
*Synechococcus* sp.						Carotenoid yield up to 0.12%		
*Dunaliella salina*						Carotenoid yield up to 1.3%		
*Nannochloropsis gaditana*	CO_2_ and 5% ethanol	Freeze drying	60	500	3	Total carotenoids 2.893 mg/g algae dw ^e^	Supercritical extraction process with co-solvent was more selective than conventional methanol extraction.	[123]
*Synechococcus* sp.			50	300		Total carotenoids 1.860 mg/g algae dw ^e^		
*Dunaliella salina*						Total carotenoids 9.629 mg/g algae dw ^e^		
*Haematococcus pluvialis*	CO_2_ and 2.3 mL/g sample ethanol	Freeze drying (powder form)	65	435	3.5	Astaxanthin recovery of 87.42% from sample containing 2.26% astaxanthin.	Increasing co-solvent amount resulted in an improving astaxanthin yield.	[121]
*Synechococcus* sp.	CO_2_ and ethanol	–	40 and 60	400 and 200	3	β-carotene 0.70 mg/g algae dw at 40 °C 400 bar ^e^ Zeaxanthin 0.70 mg/g algae dw at 60 °C 200 bar	CO_2_ with ethanol simultaneously extracted β-carotene and zeaxanthin.	[118]
*Arthrospira platensis* ^f^	CO_2_ and 26.7% ethanol	Air drying and milling	60	150	0.83	Total carotenoids 283 mg/g algae ^e^	MAE resulted in better extraction yield than SFE.	[126]
Seaweeds								
*Undaria pinnatifida*	CO_2_ and ethanol	Freeze drying	50	200	1	Fucoxanthin 7.53 mg/g dw	Yield was dependent on pressure and temperature combination.	[19]
*Undaria pinnatifida*	CO_2_	Milling and microwave-assisted cell disruption	40	400	3	Fucoxanthin 38.5 mg/g ^e^	MW pretreatment increased fucoxanthin yield.	[20]
*Sargassum muticum*	CO_2_ and ethanol	Freeze drying and comminutating	50	200	1	Fucoxanthin ~0.12 mg/g algae dw	Use of co-solvent increased fucoxanthin yield by 90 times.	[29]
*Undaria pinnatifida*	CO_2_ and 3.23% ethanol	Drying	60	400	3	Fucoxanthin 0.9945 mg/g dw ^e^	Use of co-solvent increased fucoxanthin yield by 10 times.	[23]
*Undaria pinnatifida*	CO_2_	Drying	60	400	2.5	Fucoxanthin ~0.058 mg/g dw ^e^	Pressure, temperature and extraction time affected fucoxanthin recovery.	[129]
*Saccharina japonica*	CO_2_ and ethanol	Freeze drying and grinding	45	250	2	Fucoxanthin 0.41 mg/g dw ^e^	SFE process extracted a similar content of fucoxanthin as when acetone–methanol conventional extraction was used.	[137]
*Sargassum horneri*						Fucoxanthin 0.77 mg/g dw ^e^		
*Saccharina japonica*	CO_2_ and 2% sunflower oil	Freeze drying	50.62	200	2	Total carotenoids 2.391 mg/g dw ^e^; fucoxanthin 1.421 mg/g dw ^e^	Sunflower oil as a co-solvent found to be the most effective, than soybean oil, canola oil, ethanol, and water.	[26]

^a^ Ethanol/vegetable oils mentioned in the column served as a co-solvent in the extraction; ^b^ Operating temperature; ^c^ Operating pressure; ^d^ Extraction time; ^e^ Maximal yield obtained at optimum conditions; ^f^ Considered as cyanobacteria.

**Table 3:** Comparison of different extraction techniques for extraction of lutein from *Chlorella pyrenoidosa* (reproduced with permission from [138]).

Extraction Method	Temperature (°C)	Pressure (MPa)	Ultrasound Power (W)	Time (h)	Lutein Yield (μg/g)
SE	43	0.1	0	18	546.4
SWE	150	5	0	1/3	0
SCE	50	25	0	4	393.3
SCCE	27	21	0	4	422.9
SCCE with pretreatment	27	21	0	4 (+3 h pretreatment)	921.5
USCCE with pretreatment	27	21	1000	4 (+3 h pretreatment)	1240.1

SE—Soxhlet extraction; SWE—subcritical water extraction; SCE—supercritical CO_2_ extraction; SCCE—subcritical CO_2_ extraction; USCCE—ultrasound-enhanced subcritical CO_2_ extraction; pretreatment includes enzymatic treatment with cellulose prior to extraction.

**Table 4:** Comparison of conventional and pressurized extraction techniques for recovery of fucoxanthin from *Undaria pinnatifida* (reproduced with permission from ref. [23]).

Extraction Techniques	Time (h)	Temperature (°C)	Pressure (MPa)	Fucoxanthin Yield (μg/g)
Ethanol (Soxhlet)	12	78	ND	50
Liquefied DME	0.72	25	ND	390
Supercritical CO_2_	3	60	40	60.12
	3	70	40	59.51
Supercritical CO_2_ with entrainer (3.23%)	3	60	40	994.53

ND—Not determined.

**Table 5:** Summary of ultrasound applications to enhance carotenoids from microalgae.

Microalgae	Extraction Condition								Carotenoid Yield/Recovery	Notes	Reference
	Solvent ^a^	Cell Concentration (g Cells Dry Weight/L)	Pretreatment	*f* ^b^ (kHz)	P ^c^ (W)	t ^d^ (min)	E ^e^ (kJ/kg)	T ^f^(°C)			
*Chlamydomonas reinhardtii*	Water	1.5	Frozen cells with glycerol, thawing and suspension in artificial seawater	20	2200	0.17 or 0.5 min at various amplitudes	0–450	N/A	Carotenoids—0.3 carotenoids/mg cells	91%–95% disruption; 80 kJ/kg regardless of cell concentration	[154]
*Chlorella pyrenoidosa*	Subcritical CO_2_ at 5–35 MPa	N/A	no treatment, ethanol soaking or enzymatic pretreatment	20–24	0–19 W/cm^2^; 0–1000 W 15–45 kg/h, time 0–6 h,			15–33	Lutein—87–124 mg lutein/100 g Chlorella	Ultrasound-enhanced subcritical CO_2_ extraction	[138]
*Haematococcus pluvialis*	Ethanol and ethyl acetate	50	From dried algae	40	200	10–20	120–240	30–50	Astaxanthin—27 mg/g	US led to higher astaxanthin compared with conventional treatment	[155]
*Chlorella vulgaris*	Ethanol (90%)	N/A (31 mL solid/g solvent)	With or without enzymatic pre-treatment, 50 °C	35	56 W/cm^2^	60–240	N/A	37	Lutein—3.16–3.36 mg/g wet weight	Highest ultrasound-based extraction was with enzymatic pre-treatment	[156]
*Cylindrotheca closterium (bacillariophyte)*	Acetone	N/A, 30 mL	Freeze dried	N/A	4.3–12.2	3–15	25–350	8.5	Fucoxanthin 3.5–4.5 g/mg	-	[144]
*Dunaliella salina*	Water	N/A	None	20, 580, 864 and 1146	32.3, 3, 20, 60	30	5.4	15–20	Carotenoids (yield not reported)	Inactivation efficiency 20 < 580 = 864 < 1146 kHz	[157]
*Dunaliella tertiolecta (chlorophyte)*	Water	30 mL	None	N/A	4.3–12.2	3–15	25–350	8.5	β-carotene—5 mg/g		[144]
*Haematococcus pluvialis*	Methanol, ethanol, acetonitrile, acetone	0.1 g/30 mL	None	38.5	18.4	0–90	2000	30–60	Astaxanthin—73% recovery	55%–60% yield increase of astaxanthin after US	[145]
*Spirulina platensis*	*n*-heptane, diethyl ether and hexane	10–60 g/L solvent	Spray dried spirulina mixed with methanol and kept fat various times	20	50–165 W (167 W/cm^2^)	8 min with cycling	220 kWh/m^3^	10–50	β-carotene—0.8–1.0 mg/g	Extraction had variable increase with acoustic intensity.	[158]

^a^ 1–2 mL solvent/g; ^b^ Frequency; ^c^ Power; ^d^ Time; ^e^ Specific energy; ^f^ Temperature.
